# Treating the innocent victims of trolleys and war

**DOI:** 10.1111/bioe.13273

**Published:** 2024-02-22

**Authors:** Michael L. Gross

**Affiliations:** ^1^ School of Political Science University of Haifa Haifa Israel

**Keywords:** collateral harm, compensation, just war, liability, medical rules of eligibility, military necessity

## Abstract

Both trolleys and war leave innocent victims to suffer death and injury. Trolley problems accounting for the injured, and not only the dead, tease out intuitions about liability that enhance our understanding of the obligation to provide compensation and medical care to civilian victims of war. Like many trolley victims, civilians in war may suffer justifiable, excusable, or negligent harms that demand compensation. Chief among these is collateral harm befalling civilians. Collateral harm is endemic to war and comprises permissible but unavoidable death or injury following necessary and proportionate military operations. Although state armies sometimes offer condolence payments for civilian death, injury, and property loss, they deny liability. Instead, they use compensation to enhance counterinsurgency efforts and assuage feelings of agent regret. As part of the medical rules of eligibility, Coalition forces in Iraq and Afghanistan also provided medical care to victims of collateral harm. However, they denied care to similarly sick or injured civilians. While compensation is often justified to cure the harm civilians suffer, the differential use of medical resources is not. Rather, medical care remains subject to the principle of beneficence and medical need. The duty to provide civilian healthcare in war, particularly in wars of humanitarian intervention, is far‐reaching and imposes significant costs that military and medical ethics are yet to recognize.

## 1 INTRODUCTION

It is a peculiar feature of trolley accidents that their victims never live to tell about them. Those on track A live, and those on track B die. There is a simple reason that trolleys leave no injured: trolley problems test our intuitions about causing or failing to prevent harm, and death quantifies harm most concretely. But what happens if some are injured? This raises an entirely new set of questions about the obligations the trolley driver owes to those on the track but still breathing. That this raises a parallel question during the war is obvious: What obligation do military organizations owe civilians injured during armed conflict? The answer is not obvious. There are few precedents for acknowledging any duty of redress to civilian victims of war. Nevertheless, and for myriad reasons, the United States and its allies offered cash payment and medical care to civilians directly wounded by Coalition forces during military engagements in Iraq and Afghanistan.

Under the medical rules of eligibility (MRoE), local civilians facing an imminent threat to “life, limb, and eyesight” only qualify for treatment if

*Rule 1*: Bed space is available at a combat support hospital that is not needed to treat multinational forces and

*Rule 2*: Their injuries are collateral, that is, the direct result of Coalition action.^1^

The MroE only admit collaterally injured civilians to Coalition combat support hospitals for emergency care. Once stabilized, they transition to the local healthcare system for continued treatment. No other ill or injured civilians are eligible for admission. The MRoE are not a legal imperative. Combat exclusions turning on national sovereignty, customary international humanitarian law (IHL), and attendant financial costs specifically exempt military forces from legal liability when their soldiers collaterally kill civilians during combat operations.^2^ For these reasons, the parties to an armed conflict historically care solely for their own. Morally, however, the rules are vexing. If collateral harm is morally permissible, what claim do its victims have against Coalition forces?

One answer is that we owe them nothing if the harm they suffer is entirely justified. Another answer recommends compensation or amends^3^ because collateral deaths are never justified but, at best, only excused and, at worst, the result of negligence. A third response affirms the benefits of compensation because it appeases victims' anger, offers significant military and political benefits, and assuages injurers' feelings of agent regret and moral injury.

Whether such care is appropriate depends upon how we morally categorize wartime collateral harm. As a prelude, trolley problems illustrate examples of justifiable, excusable, or negligent harm. Translating these cases into wartime medicine challenges our intuitions about who deserves what kinds of compensation and why.^4^ Moreover, they set the stage for understanding whether and how it is proper to use medical care to compensate civilians for collateral harm. In Iraq and Afghanistan, Coalition forces provided money and medicine, and while the financial payment is appropriate, offering scarce medical resources is considerably more complex.

## 2 JUSTIFIABLE, EXCUSED, AND NEGLIGENT HARM IN TROLLEYS AND WAR

Trolleys are not weapons, but they tease out our moral intuitions about who deserves what kind of compensation when they injure those stuck on the tracks.

### 2.1 Trolley injured and maimed

Consider the following cases:

1. *Justified killing*: There are five people on Track A and three people on Track B, and the trolley driver^5^ diverts the trolley to Track B. One person dies, and two are injured on Track B. All on track A live.
2. *Excused killing*: There are five people on Track A and three people on Track B, and the driver diverts the trolley to Track B. But, the lighting is poor, and the people are standing in such a way that he underestimates the number of people on track B. There are, in fact, six people on Track B, of whom three die and three are injured. The five on Track A survive.
3. *Negligent killing*: Five people are on Track A and three on Track B, and the trolley driver tries to divert the trolley to an empty track. However, the controls malfunctioned due to an obstruction the driver should have noticed during his required predrive inspection. The trolley continues to Track A, where four die and one is injured.

In each case, liability for compensating those harmed looks to the driver's moral state and the status of the victims' rights. To incur fault, the injurer (driver) must (1) “fail to satisfy a standard of reasonable conduct, and (2) in some sense that failure must be imputable to him as his doing.^6^” Condition (1) denotes wrongdoing, and condition (2), responsibility. Each also affects the status of the victim's rights. Negligent harm comprises both wrongdoing and responsibility and, thereby, *violates* the victim's rights, leaving the injurer liable for punishment and payment of compensation. Excused harm, however, acknowledges wrongdoing but denies responsibility, as might be the case if the injurer is coerced or, in this case, subject to unanticipated lighting. Here too, the victim's rights are violated, so the driver is liable for compensation but often not punishment. In contrast, justified harms carry neither wrongdoing nor responsibility. Justified self‐defense is one case where the defender injures his attacker but incurs no liability because the attacker has forfeited his rights. In trolley case (1), the injuries suffered by those on track B were justified. As a result, the driver‐injurer did not forfeit his rights, nor were the injured wronged. Nevertheless, their rights were *infringed* (not violated) when they suffered losses they did not deserve. The driver, therefore, owes the injured some level of compensation. However, if liability for excused acts is weaker than negligent liability, then liability for justified acts is weaker still.^7^

How does this logic play out in war?

### 2.2 Collateral harm in war

Collateral or incidental harm falls on any civilian noncombatant killed or injured as the unintended but unavoidable result of a necessary and proportionate military operation. The harms are incidental because they serve no military purpose. As a prelude to mapping the obligation to care for civilians, the following cases exemplify justified, excused, and negligent harm.

1a. *Justified collateral harm*
  Using precision‐guided munitions, preplanned attacks on leadership, government, and security buildings in Baghdad in 2003 led to few known civilian casualties. Weapon choice and accuracy, detailed collateral damage assessments, and fusing contributed to the low casualty rate from bombing.^8^
2a. *Excused collateral harm*
  Responding to a call by German forces in Kunduz in 2009, an American F‐15E fighter jet struck two fuel tankers captured by Taliban insurgents, killing over 90 civilians. German forces stated that the strike occurred after an unmanned surveillance aircraft had determined that there were no civilians in the area, and commanders believed that only insurgents were in the vicinity.^9^
3a. *Negligent harm*
  The Pentagon maintained that the deadly U.S. strike on an MSF hospital in Kunduz, Afghanistan, in 2016 that killed 42 people resulted from unintentional human error and equipment failure.^10^

Consistent with the previous discussion and despite laws excluding armed forces from legal liability, the United States of America and Germany paid up to $5000 to each victim or their families when they suffered negligent or excusable harm (Cases 2a and 3a). Offers of medical care were not documented but certainly justified by the same argument. However, the case of justified collateral harm (Case 1a) is more complex.

Prior to the wars in Iraq and Afghanistan, there were few documented cases or discussions surrounding the moral obligation to compensate victims of collateral harm or offer anything but ad hoc medical care to local civilians. Case 1a represents the gold standard of collateral harm where sufficient time allows planners to carefully calculate proportionality and choose targets to avoid “incidental loss of civilian life, injury to civilians, damage to civilian objects, or a combination thereof, which would be excessive in relation to the concrete and direct military advantage anticipated.^11^” This is precisely what international law and just war theory demand. If so, then planners are blameless, without wrongdoing or responsibility, and not morally liable to make restitution. That victims of collateral harm are owed nothing seemed so obvious; few theorists question this conclusion.^12^ However, the argument is suspect because collateral harm infringes on the victims' rights. It is, therefore, noteworthy that Coalition forces made cash “condolence” payments and provided medical care to civilians injured as the direct result of U.S. and Coalition operations in Iraq and Afghanistan.^13^ This policy is a radical departure from warfighting but is only obligatory if collateral harm recognizes that Coalition soldiers, albeit blamelessly, infringed upon victims' rights. Because compensation depends upon the extent of wrongdoing, the duty to compensate victims of collateral harm is weaker than that due to victims of excusable or negligent harm. Judging by the trolley examples as well as the propensity of soldiers in the field to seek monetary compensation, their duty is not insignificant.

Justification for financial compensation lies in liability. But why do armed forces suddenly recognize liability for collateral harm where there previously was none? Several answers, from military necessity to agent regret and moral injury, suggest themselves. Whether these extend to privileged access to medical care is a question the subsequent discussion raises.

## 3 RECOGNIZING THE DUTY TO COMPENSATE

Very different reasons turning on military necessity, agent regret, and moral injury may cause armies to compensate for collateral injuries.

### 3.1 Military necessity

Searching for insurgents in Iraq in late 2003, U.S. troops sometimes raided the wrong house and killed or injured innocent civilians. In time, they learned that such errors were compensable: “(Brigade commander Col David) Hogg began to understand that when you make a mistake, you apologize, explain how it occurred, and give the householder one hundred dollars.”^14^ As compensation guidelines developed, U.S. and Coalition forces agreed to make ex gratia “condolence” or “solatia” payments to civilians harmed as the direct result of U.S. and Coalition operations in Iraq and Afghanistan. At the same time, however, they assiduously avoided any admission of liability. Compensation refers only to a payment or service for a loss and includes cash payments from $2500 to $5000 for each loss of life, injury, or property, as well as medical care for the injured.^15^

Support for condolence payments draws on military necessity and sidesteps the question of liability. Condolence payments and accompanying medical care were a “politically prudent step to avoid alienating the population from troops” and “designed primarily to quell anger among the local population” in a culture that accepts payment for accidental or unintentional loss of life.^16^ Coalition planners anticipated that payments would aid counterinsurgency efforts by making it difficult for insurgents to gain support and recruit fighters from among the local population.^17^ The appeal to military necessity ignores liability and invokes the benefits that compensation offers the *injuring* party. It is, therefore, situationally dependent. Israel, for example, has had little reason to think that compensation might win Palestinian hearts and minds any more than Allied forces thought they might convince German or Japanese civilians to defect to the Allied cause. The lack of military benefits removes the incentive to provide “condolence” or “solatia” payments to civilians harmed collaterally.

### 3.2 Agent regret and moral injury

Unlike military necessity, agent regret and moral injury tie the injurer to feelings of liability. In Bernard Williams' paradigm case, a driver suffers “agent regret” or remorse after accidentally killing a child who suddenly darts into the street.^18^ In war, moral injury is the pathological cousin of agent regret and reflects deep feelings of non‐culpable responsibility.^19^ Like agent regret, moral injury turns on the agent's causal role in another's death or injury, irrespective of its justification or excuse. Morally injurious acts include killing or contributing to the deaths of combatants or noncombatants or failing to prevent such deaths. Such acts are often justified, excused, or blameless. Affecting 12%–18% of returning U.S. troops, moral injury exacts a severe psychological and emotional toll, including guilt, shame, feelings of worthlessness, self‐condemnation, poor self‐esteem, and other symptoms that overlap with post‐traumatic stress disorder.^20^

Agent regret is a vexing response in these situations because the soldier (or driver) has committed no wrong and is not at fault. Agent regret results from the driver's (or soldier's) pivotal role in the child's (or the civilian's) death. It is the thought, “How much better if it had been otherwise.”^21^ Agent regret is the agent's expression of sorrow and compunction despite blamelessness and demonstrates, as Williams notes, that we cannot “entirely detach ourselves from the unintentional aspects of our actions… and yet still retain our identity and character as agents.”^22^

In war, agent regret and moral injury sometimes follow the killing of combatants.^23^ This phenomenon seems unusual because killing a combatant is entirely justified, and liability is, therefore, nonexistent. If a telltale sign of agent regret is the hope that things had been otherwise, then most soldiers would not (indeed, should not) hope for a different outcome. There is no prima facie case for one combatant to pay compensation to another. Killing noncombatants is considerably more disturbing. Here, the hope that things had been otherwise is indeed relevant because collateral, noncombatant deaths offer no military advantage. Agent regret may explain why some agents feel liable for the harm they cause and wish to pay compensation. These sentiments were prominent in a case similar to 2a, where German soldiers killed a woman and two children who failed to stop at a checkpoint in Afghanistan in 2008.^24^ Here, civilians suffered excusable harm that saddled the soldiers with moderate liability to pay compensation. Recognizing their role and wishing things might have been otherwise, the soldiers successfully petitioned for a sizeable financial award for the families.

Military necessity, agent regret, and recognition of liability are reasonable grounds for compensating victims of war. Firm norms, however, remain weak. Military necessity will not dictate compensation in all cases. Domestic law regulating military organizations continues to deny liability for combat‐related deaths, and not all soldiers or trolley drivers suffer agent regret or moral injury. Given the norms of medical ethics, however, might a stronger case be made for compensatory medical care for civilians in war?

## 4 MEDICAL CARE AS COMPENSATION FOR COLLATERAL HARM?

If victims of collateral harm are entitled to financial compensation, it also seems they are due medical care for their injuries. Yet consider the following case.

Case 4. *The trolley and the automobile*: There are five people on Track A and three people on Track B; the driver diverts the trolley to Track B. 1 person dies, and two are injured. At the same time, a car crashes into a tree just next to the trolley. In the car crash, one person dies, and two are injured. The trolley driver grabs a first‐aid kit and runs out to aid the injured.

Case 4 asks the comparative question. Whom should the trolley driver aid first? Do the trolley victims have a greater right to the driver's attention than the car accident victims? On the one hand, the answer is no. As a utilitarian‐based rule, beneficence demands that the trolley driver helps as many individuals as possible. If the conditions of the accident are such that she can attend to the automobile passengers more effectively than the trolley victims, then she should treat them first, irrespective of the circumstances surrounding each case. Yet, if we follow the logic of compensation, the driver must attend first to those whose rights she infringed by harming them. They have a stronger claim to her aid than the random victims of an automobile accident.

To sharpen the dilemma, consider medical care during war:

4a. Medical Care for Afghan Civilians (May–November 2011).

In the days following a firefight between Coalition forces and Taliban fighters, sick and injured Afghan civilians sought treatment from a Coalition forward surgical team (FST). Among the patients were those suffering collateral injuries of the battle (e.g., burns or fractures) and those suffering from unrelated work accident injuries and disease.^25^ Faced with limited resources, the FST commander approved the treatment of the battle‐injured civilians but not the others.

Which moral rules or intuitions guide us here as the medical duty of beneficence collides with the duty to compensate those whose rights we infringe? Agent regret and military necessity figure prominently, but do not easily reconcile with the duties of beneficence and the norms of medical impartiality.

### 4.1 Military necessity and agent regret

In war, medical care has significant military value. Clearly articulated by the phrase “medical diplomacy,” the judicious and strategic use of medical care can win hearts and minds and influence the local population to support a government at war.^26^ Although the medical rules of eligibility formerly excluded patients who were not injured by Coalition fire, hospital commanders often found it expeditious to admit sick civilians or those injured in accidents or by insurgents when bed space was available.^27^ Apart from emergency trauma cases, burns, and fractures, hospitals provided neurosurgery, craniofacial repair, and pediatric care.^28^ Medical Civic Action Programs also operated mobile clinics. In return, many medical personnel and administrators expected that sophisticated medical care, particularly pediatric care, would build trust, generate goodwill, strengthen support for the government, and bring actionable intelligence about insurgent plans and capabilities.^29^ Military necessity, however, does not speak directly to victims of collateral harm but to anyone who can provide some military benefit. “Medical stability operations,” for example, targeted insecure and insurgent‐threatened areas and provided medical care to pacify the civilian population and mobilize domestic backing for the national government.^30^

Although medical stability operations emphasize military necessity, questions about liability were not lost. “If we hurt them, we fix them” was a common sentiment among Coalition medical personnel.^31^ On its face, it evokes the liability inherent in agent regret and the obligation to make amends simply because one harmed another, however justifiable or excusable. In these ways, liability ignores beneficence to focus on the antecedent conditions of one's injury, just as medical diplomacy disregards beneficence to count the military advantages of medical care. In doing so, each justifies differential care for identically sick or injured parties. But neither social nor military benefits nor liability are normally acceptable healthcare criteria. Doctors and nurses do not only fix those they (or their compatriots) hurt. Instead, healthcare is, or should be, oblivious to the circumstance and social implications of illness or injury and focus solely on medical needs. “If we hurt them, we fix them” might be a laudable reaction in one sense (because it embraces care for oft‐neglected local civilians), but it also excludes large swaths of sick and injured who suffer from injuries unrelated to Coalition operations. Indeed, the principles of impartiality and beneficence repudiate any consideration of patient fault when delivering medical care. Those at fault for their injuries (e.g., drunk drivers) are no less entitled to care than those they hurt. How, then, does fault‐based care during war square with the ethical imperative of impartiality?

### 4.2 Medical impartiality, beneficence, and liability

Trolley and collateral victims of war call attention to the complexity of compensating victims of permissible collateral harm with medical care. While cash compensation addresses liability by trying to make victims whole, medical care also answers the victim's right to rescue. The right of rescue draws from the duty to rescue those in need by those in a position to tender aid at some reasonable cost to themselves.^32^ The meaning of “reasonable cost” is subject to varying interpretations but is exceptionally high among medical professionals pursuant to their sworn duties of beneficence. Beneficence reflects a “moral obligation to act for the benefit of others.”^33^ “Benefit” unpacks to include saving lives and/or improving their quality. For those enjoying the right to rescue, medical impartiality demands that caregivers treat patients solely based on medical need and without any distinction related to combatant status, national identity, gender, race, etc., an obligation that international humanitarian law enshrines in the Geneva Conventions.^34^ On the other hand, this principle is not absolute. Rules of mass casualty triage bow to military necessity as they obligate caregivers to return compatriot soldiers to battle before treating more critically wounded compatriot or enemy patients.^35^ The medical rules of eligibility are not medically neutral and endorse preferential care for some compatriots regardless of others' needs. Might then liability temper the reach of impartiality and beneficence? Consider three variations of trolley problem 4:

*Tie‐breaking*: Equal numbers of severely injured trolley and automobile victims litter the scene. Here, the trolley driver invokes agent regret and/or weak liability to break the tie and treat the trolley victims first.

*Minor Loss*: Following the two accidents, five severely injured automobile victims outnumbered four trolley victims. Here, the trolley driver invokes agent regret and/or liability to offer additional grounds to treat the trolley victims first. Because of his actions, all the trolley injured lived, but a slightly greater number of automobile victims died.

*Major Loss*: Following the two accidents, ten severely injured automobile victims significantly outnumber four trolley victims. Here, the trolley driver invokes agent regret and/or liability to offer additional grounds to treat the trolley victims first. Because of his actions, all the trolley injured lived, but all ten of the automobile victims died.

Morally acceptable tiebreakers include a lottery or “first come, first served,” which in emergencies is needlessly distracting and time‐consuming. Instead, the driver immediately acts on agent regret and intuitions of liability to tip the scales in favor of the trolley victims. The question is: “How much heavy lifting can liability perform before falling to the duty of beneficence?” As far as I know, medical ethics has yet to address this question and might easily say none; the principle of beneficence makes no room for liability any more than it makes room for antecedent conditions relating to a patient's character, moral shortcomings, or bad luck. All are entitled to care on the sole basis of medical need. Still, it is hard to ignore the call of “if we hurt them, we fix them,” permitting the driver to attend to the trolley wounded, all things being equal.

In practice, however, all things are rarely equal. Minor and Major Loss scenarios are the norm. In *Minor Loss*, the duty to aid others and save as many lives as possible should point him toward the larger group. As he considers his options, all kinds of considerations may play out. To what extent was he responsible? What does he owe the various injured? Time may prevent a full assessment, but his concerns are not trivial. While excused behavior suggests limited liability, negligent actions suggest something more substantial. If the trolley driver fell asleep and only woke up to the screams of wounded on the track, is his liability to tender aid to them thereby stronger? He might think so and offer this as a reason for aiding the fewer trolley victims first. But at one point, the call of liability wears thin if the needs of the automobile victims are significantly greater than those of the trolley victims, as in *Major Loss*. And, if beneficence is decisive when automobile victims greatly outnumber trolley victims, it might be equally decisive when the automobile victims only slightly outnumber trolley victims (*Minor Loss*).

In war, however, there is some leeway here. Judging by norms of triage, beneficence rules unless there is overriding evidence of a significantly greater non‐medical benefit from a different distribution of medical resources. One would not permit medical personnel to abandon the seriously injured unless absolutely necessary to save the day. To override impartiality and offer privileged care to victims of collateral harm would require firm evidence, currently lacking, of a significant benefit money cannot buy.^36^

Nevertheless, medical care can minimize bodily harm in a way that money cannot, thereby addressing an overlooked aspect of military necessity. Military necessity strives to minimize costs and, indeed, minimizing harm is a feature of many interpretations of proportionality.^37^ Along these lines, financial compensation may aid large numbers of civilians suffering life‐threatening diseases and injuries.^38^ However, personal cash compensation cannot easily rectify collateral and other injuries or illnesses. Instead, communities can only restore health and well‐being with a massive infusion of post‐war reconstruction funding to rebuild health, agricultural, and sanitation infrastructures.^39^ And, while postwar aid is often due to the victims of conflict, military planners may ask a more direct question at the point of collateral injury during the war: “Can our forces immediately minimize harm by evacuating and treating civilian casualties?”

Such an interpretation constitutes a major change in the calculation of proportionality that does not ordinarily require an army to figure in medical care for local civilians. In this context, however, minimizing harm means minimizing harm *overall*, so consideration of other costs—the greater risk that comes with evacuating civilians and/or the material cost of providing additional military personnel and medical resources, for example—is relevant. When minimizing harm overall is enhanced by providing medical care to civilian casualties without imposing prohibitive costs that might hamper any army's war effort, there are solid grounds for aiding victims of collateral harm independent of military advantage. Including medical care in proportionality calculations, moreover, might also prove advantageous if it permits a more forceful military operation than a calculation absent medical care would allow. On the other hand, the financial and logistical ramifications of providing medical care to all wounded civilians whose rights were infringed or violated may easily overwhelm a military healthcare infrastructure designed and funded to treat and evacuate warfighters. At the height of the war in Iraq, for example, the United States only maintained 274 operational beds to serve 150,000 deployed troops between 2006 and 2008.^40^

The case for compensation, therefore, plays out differently in peace and war. Trolley problems, for example, cannot invoke military necessity to justify financial compensation or medical care. Nevertheless, trolley problems usefully direct our attention to how liability can affect the duty to care. *Case 4* is particularly vexing. For some readers, the driver's duty to first attend to the trolley wounded is obvious. In contrast, international humanitarian law and principles of impartiality and beneficence reject any attempt to consider differential entitlements for victims of collateral harm compared to those suffering other injuries or diseases. In the final analysis, the grounds for compensation are fluid and variable but never entirely absent from modern war or runaway trolleys. Trolley victims invoke the liability of their injurers, while victims of collateral harm may appeal to the interests or conscience of their injurers. What resources, then, are best suited to compensation?

## 5 THE COIN OF COMPENSATION: MONEY OR MEDICINE?

Compensating victims of collateral harm in war is a novel enterprise with little attention from military law and ethics. Compensation is primarily monetary. Whether $2500 or $5000 is sufficient is a difficult question to resolve. Coalition forces suggest this was enough to remedy the loss of life, injury, or property destruction under local custom. Negotiations with local leaders sometimes brought larger payments but attempts to sue the German government for more than $5000 in *Case 2a* (excused collateral harm) were rejected because a German court found no evidence of dereliction of duty. Overall, condolence payments were not a significant military expense. For example, the U.S. military disbursed about 7M dollars in Afghanistan from 2005 to 2019, covering roughly 20% of those eligible.^41^ Condolence payments in Iraq totaled 21M dollars in 2005 and 7M in 2006.^42^

These amounts hardly seem sufficient. The plight of trolley wounded suggests firm grounds for compensation as the harm they suffer moves from justified to excusable to negligent, and as hard rights violations replace softer rights infringements. Ordinarily, the aggrieved merit financial compensation, but injured and suffering individuals are entitled to something more to heal their wounds. Medical care, however, is rarely sensitive to liability when others are in equal or greater need. Nevertheless, the appeal to liability is sometimes so overwhelming that ignoring the cause of one's injury is tantamount to further abuse. A trolley driver who gets drunk and falls asleep may not meet this threshold, but perpetrators of war crimes might. One might, therefore, demand that German doctors treat Holocaust victims before local citizens severely injured by Allied bombing. The example is vivid but not unique. Armed forces intervening on behalf of a severely persecuted ethnic or religious group might impose similar demands on local medical providers.

For all its novelty, wartime compensation leans heavily on military necessity, a principle intrinsic to the conduct of war. Military planners contemplating condolence payments or formulating medical rules of eligibility need not stray far from their comfort zone. But their reach is limited. Coalition forces did not compensate all they might or should. Condolence payments were often symbolic gestures to make amends as part of a broader public diplomacy policy. Medical care, too, found its place in the context of medical diplomacy but was often so inadequate or intermittent to be both poor medicine and poor diplomacy.^43^ Unanchored in any concise guidelines to satisfy military necessity, condolence payments and medical care were often ad hoc.

That trolley‐roused intuitions about compensation do not underlie wartime thinking does not mean that they should not. If, as some independent observers in Afghanistan concluded, “an apology and compensation do appear to alleviate the anger and resentment of many Afghan civilians,” then an effective policy might establish a permanent institutional mechanism to adjudicate combat‐related claims and tacitly acknowledge liability far more than Coalition forces have done.^44^ Treating sick and injured civilians, however, is far more complex. Beyond liability, additional factors, including the narrow duty to minimize casualties and avoid disproportionate harm and the broader duty to attend to all civilians solely based on medical need, drive the obligation to provide medical care. Moreover, the duties to care for civilian victims of armed conflict differ significantly in defensive and humanitarian wars.

In a defensive war against aggression, collateral harm is a lesser evil. The greater evil is to suffer defeat, invasion, and occupation. The moral imperative to minimize collateral harm and care for enemy civilians is subordinate to the greater goal of national defense and self‐preservation. Enemy civilians, therefore, enjoy the right to rescue only if adequate resources are available for their care. Otherwise, the cost of rescue may not be sufficiently reasonable to trigger the duty. Consider a Ukrainian attack that kills and injures Russian civilians in that conflict. Insofar as these casualties are not disproportionate, liability remains weak and subject to resources not needed for Ukrainian defense. Israel's 2023 war against Hamas offers similar lessons. Civilian deaths, reported at over 20,000 by February 2024, were extremely heavy as Israel sought to neutralize missile attacks and remove Hamas from power.^45^ While the lion's share of responsibility lies with the aggressor, in this case, Hamas, the defending nation's liability to address collateral injuries and deaths is not negligible. Condolence payments and medical care can satisfy this duty. Fighting to topple Hamas, Israel also acquires an incentive to win Palestinian hearts and minds to galvanize support for the new postwar government that Israel hopes will govern Gaza. To this end, Israel can erect the mobile field hospitals it maintains for disaster relief and deploy them to Gaza. Israel can also consider reprioritizing discretionary spending to make condolence payments.

In contrast to defensive war, humanitarian interventions are justified by the claim rights persecuted people hold against wealthier nations best positioned to rescue and protect them. On this account, only egregious persecution suffered by Iraqi and Afghan civilians at the hands of Saddam Hussein and the Taliban could justify Western efforts to wage war on their behalf. The goal of a humanitarian war is not national self‐preservation but to improve the welfare of the target population. Infused with the obligation to rescue the local population from oppression and ensure their welfare, humanitarian wars generate greater concern for the victims of war than defensive wars do. The obligation to attend to the welfare of the civilian population does not stem from liability but from the comprehensive duty of beneficence, rescue, and protection. As a result, medical care for local nationals is a very high priority and an integral part of the military mission. Going to war with anything short of what is required to attend to their well‐being and minimize collateral harm would constitute an egregious violation of the soon‐to‐be victims' rights. And although collateral harm is an inescapable feature of war, a justified war of humanitarian intervention should not leave the intended beneficiaries worse off when the fighting stops. To this end, military interventions and postconflict reconstruction require considerable medical resources that raise the cost of war and demand expenditures that states, international humanitarian law, and military ethics are only beginning to acknowledge.

## Footnotes

1 Ministry of Defence (UK). (2014). *The Military Medical Contribution to Security and Stabilisation*, (JDN 3/14), Ministry of Defence, §2.6‐2.8. https://www.gov.uk/government/uploads/system/uploads/attachment_data/file/324637/20140616‐JDN_3_14_Med_contr_DCDC.pdf; *US Army Field Manual* FM 4‐02 2013: §1–43. https://www.globalsecurity.org/military/library/policy/army/fm/4‐02/fm4‐02‐2013.pdf; Bricknell, M. C. M. (2014). Military medical contribution to indigenous (military and civilian) health sector development (HSD) within security and stabilisation operations. In J. M. Ryan et al. (Ed.), *Conflict and catastrophe medicine* (pp. 451‐475). Springer; Gross, M. L. (2021). *Military medical ethics in contemporary armed conflict: Mobilizing medicine in pursuit of just war*. Oxford University Press.

2 Walerstein, J. (2009). Coping with combat claims: an analysis of the foreign claims act's combat exclusion. *Cardozo Journal of Conflict Resolution*, *11*, 319–351; Center for Civilians in Conflict (CIVIC). (2010). *United States military compensation to civilians in armed conflict*. https://civiliansinconflict.org/wp‐content/uploads/2017/09/CENTER_Condolence_White_Paper_2010.pdf.

3 Amends refers the “practice of of warring parties providing recognition and assistance to civilians they harm within the lawful parameters of their combat operations. “Compensation” refers to “payment for loss.” CIVIC. (2023). *Amends and reparations for civilian harm in armed conflict* (p. 6). Center for Civilians in Conflict. https://civiliansinconflict.org/wp‐content/uploads/2023/01/CIVIC_Amends_Brief.pdf.

4 Amends refers to the “practice of warring parties providing recognition and assistance to civilians they harm within the lawful parameters of their combat operations. “Compensation” refers to “payment for loss.”; CIVIC, op. cit. note 3, p. 6.

5 Ordinarily, the driver is not the agent that diverts the trolley but a bystander. The trolley driver, if there is one, does not control the car. With the driver in control, he is also in a position to tender aid to the injured, which a faraway bystander is not.

6 Coleman, J. L. (1976). The morality of strict liability. *William and Mary Law Review*, *18*, 259–286, at p. 270; Also: Greenawalt, K. (1984). The perplexing borders of justification and excuse. *Columbia Law Review*, *84*, 1897–1927.

7 This discussion of rights violation and infringement draws on Feinberg, J. (1978). Voluntary euthanasia and the inalienable right to life. *Philosophy and Public Affairs*, *7*, 93–123; Rodin, D. (2011). Justifying harm. *Ethics*, *122*, 74–110, at p. 86; and Thomson, J. J. (1985). The trolley problem. *The Yale Law Journal*, *94*, 1395–1415.

8 Human Rights Watch (HRW). (2003). *Off target: The conduct of the war and civilian casualties in Iraq* (p. 50). https://www.hrw.org/report/2003/12/11/target/conduct‐war‐and‐civilian‐casualties‐iraq.

9 Associated Press. (2016, October 6). The German court: No further compensation for relatives of Afghan airstrike victims. *Military Times*. http://www.militarytimes.com/articles/german‐court‐no‐further‐compensation‐for‐relatives‐of‐afghan‐airstrike‐victims

10 U.S. Central Command. (2016). *Summary of the Airstrike on the MSF Trauma Center in Kunduz, Afghanistan on October 3, 2015*. http://www.humanrightsvoices.org/assets/attachments/documents/Oct‐3‐2015‐Kunduz‐Trauma‐Center‐Strike.‐CENTCOM‐Summary‐Memo.pdf.

11 Protocol Additional to the Geneva Conventions of 12 August 1949, and Relating to the Protection of Victims of International Armed Conflicts (Protocol I), of June 8, 1977 (API) §51.5.

12 Among them are Bazargan‐Forward, Saba. “Compensation & Proportionality in War.”; Ohlin, J. D., May, L., Finkelstein, C. (Ed.). (2017). *Weighing lives in war*. (pp. 173–187). Oxford University Press. https://doi.org/10.1093/oso/9780198796176.001.0001 and Crawford, N. (2013). *Accountability for killing: Moral responsibility for collateral damage in America's post‐9/11 wars*. Oxford University Press.

13 Money as a Weapons System (MAAWS). (2009). *MNC‐I cJ8 SOP*. Multinational Corps Iraq. 20, B‐11. https://info.publicintelligence.net/MAAWS%20Jan%2009.pdf.

14 Ricks, T. E. (2006). *Fiasco: The American military adventure in Iraq, 2003 to 2005* (p. 255). Penguin Publishing Group.

15 MAAWS, op. cit. note 13; McNerney, M., Tarini, G., Sudkamp, K. M., Lewis, L., Grisé, M., Moore, P. (2022). *U.S. Department of Defense Civilian Casualty Policies and Procedures: An Independent Assessment*. Rand Corp. https://www.rand.org/pubs/research_reports/RRA418‐1.html; US Department of Defense. (2022). *Interim regulations for condolence or sympathy payments to friendly civilians for injury or loss that is incident to military operations* https://media.defense.gov/2020/Jun/23/2002320314/‐1/‐1/1/INTERIM‐REGULATIONS‐FOR‐CONDOLENCE‐OR‐SYMPATHY‐PAYMENTS‐TO‐FRIENDLY‐CIVILIANS‐FOR‐INJURY‐OR‐LOSS‐THAT‐IS‐INCIDENT‐TO‐MILITARY‐OPERATIONS.PDF.

16 Campaign for Innocent Victims in Conflict (CIVIC). (2009). *Losing the people, the costs and consequences of civilian suffering in Afghanistan* (p. 75 and 77). https://civiliansinconflict.org/publications/research/losing‐people‐costs‐consequences‐civilian‐suffering‐afghanistan/. Also, Holewinski, S. (2012). Making amends: A new expectation for civilian losses in armed conflict. In D. Rothbart, K. V. Korostelina, and M. D. Cherkaoui (Eds.), *Civilians and modern war: Armed conflict and the ideology of violence* (pp. 317–333) Routledge.

17 Money as a Weapons System (MAAWS). (2009). *Handbook, commander's guide. 09‐27*. US Army. https://www.usma.edu/cnrcd/siteassets/sitepages/government%20publications/call%20maaws%20handbook%2009‐27%20(april%2009).pdf; Olsthoorn, P., Bollen, M., and Beeres, R. (2013). Dual loyalties in military medical care—Between ethics and effectiveness. In H. Amersfoort, et al. (Eds.), *Moral responsibility and military effectiveness* (pp. 79–96). TMC Asser Press.

18 Williams, B. (1981). *Moral luck* (pp. 20–39). Cambridge University Press.

19 Sherman, N. (2015). *Afterwar: Healing the moral wounds of our soldiers*. Oxford University Press.

20 Nazarov, A., Jetly, R., McNeely. H., Kiang, M., Lanius, R., & McKinnon, M. C. (2015). Role of morality in the experience of guilt and shame within the armed forces. *Acta Psychiatrica Scandinavica*, *132*, 4–19.

21 Williams, op. cit. note 18, p. 27.

22 Ibid: 29.

23 Maguen, S., Metzler, T. J., Litz, B. T., Seal, K. H., Knight, S. J., & Marmar, C. R. (2009). The impact of killing in war on mental health symptoms and related functioning. *Journal of Traumatic Stress*, *22*(5), 435–443.‏ https://doi.org/10.1002/jts.20451

24 Deutsche Welle 2008.

25 Woll, M., & Brisson, P. (2013). Humanitarian care by a forward surgical team in Afghanistan. *Military Medicine*, *178*, 385–388.

26 Gross, M. L. (2021). *Military medical ethics in contemporary armed conflict: Mobilizing medicine in pursuit of just war* (pp. 204–225). Oxford University Press.

27 Richardson, M. C. (2008). The complexity of moving patients in today's maturing counterinsurgency environment: Whom, when, and how. *US Army Medical Department Journal*, *October*, 41–51; Weeks, S. R., Oh, J. S., Elster, E. A., & Learn, P. A. (2018). Humanitarian surgical care in the US military treatment facilities in Afghanistan from 2002 to 2013. *JAMA Surgery*, *153*(1), 84–86.

28 Bonnet, S., Gonzalez, F., Poichotte, A., Duverger, V., & Pons, F. (2012). Lessons learned from the experience of visceral military surgeons in the French role 3 medical treatment facility of Kabul (Afghanistan): An extended skill mix is required. *Injury*, *43*(8), 1301–1306; ‏Schulz, C., Kunz, U., & Mauer, U. M. (2012). Three years of neurosurgical experience in a multinational field hospital in northern Afghanistan. *Acta Neurochirurgica*, *154*(1), 135–140; Neff, L. P., Spinella, P. C., Azarow, K. S., & Jafri, M. A. (2017). The pediatric patient in wartime. In M. J. Martin, A. Beekley, & M. Eckert (Eds.), *Front line surgery* (pp. 543–562). Springer International Publishing.

29 Bolt, J. D., & Schoneboom, B. A. (2010) Operative splenectomy for treatment of homozygous thalassemia major in Afghan children at a US military hospital. *AANA Journal*, *78*(2), 129–133; Porta, C. R., Robins, R., Eastridge, B., Holcomb, J., Schreiber, M., & Martin, M. (2014). The hidden war: Humanitarian surgery in a combat zone. *The American Journal of Surgery*, 207(5), 766–772; Gross, op. cit. note 26, pp. 214–215.

30 Baker, J. B. (2010). The doctrinal basis for medical stability operations. *Military Medicine*, *175*(1), 14–20.

31 Hodgetts, T. J., Mahoney, P. F., Mozumder, A., & Mclennan, J. (2005). Care of civilians during military operations. *International Journal of Disaster Medicine*, *3*(1–4), 3–24.

32 Weinrib, E. J. (1980). The duty to rescue. *The Yale Law Journal*, *90*, 247–293; Menlowe, M. A. (1993). The philosophical foundations of the duty to rescue. In M. A. Menlowe & A. McCall Smith (Eds.), *The duty to rescue: The jurisprudence of aid* (pp. 5–54). Dartmouth Publishing; Miller, D. (2020). The nature and limits of the duty of rescue. *Journal of Moral Philosophy*, *17*(3), 320–341; Weinrib, E. J. (1980). The duty to rescue. *The Yale Law Journal*, *90*, 247–293.

33 Beauchamp, T. L., & Childress, J. F. (2009). *Principles of Biomedical Ethics* (6th ed., pp. 197). Oxford University Press.

34 API (1977), §10.

35 Gross, M. L. (2006). *Bioethics and armed conflict, moral dilemmas of medicine and war* (pp. 137–174). MIT Press.

36 McInnes, C., Rushton, S. (2014). Health for health's sake, winning for god's sake: US global health diplomacy and smart power in Iraq and Afghanistan. *Review of International Studies*, 40, 835–857.

37 I thank Paul Gilbert for first raising this point at an ICMM meeting (International Committee on Military Medicine) in May 2017.

38 Bazargan‐Forward, Saba. (2017). Compensation & proportionality in war. Such “indirect” casualties have been estimated at 4–10 times greater than direct casualties. See: Geneva Declaration Secretariat. (2015). *Global burden of armed conflict*. 43 (n. 19). http://www.genevadeclaration.org/fileadmin/docs/GBAV3/GBAV3_Ch1_pp11‐48.pdf and Wise, P. H. (2017). The epidemiologic challenge to the conduct of just war: confronting indirect civilian casualties of war. *Daedalus* 146(1), 139–154.

39 Gross, op. cit. note 1, pp. 229–253.

40 Richardson, M. C. (2008). The complexity of moving patients in today's maturing counterinsurgency environment: Whom, when, and how. *US Army Medical Department Journal*, *October*, 41–51.

41 McNerney, op. cit. note 15. However, this report describes 2478 condolence payments while the deaths attributed to Coalition forces stood at 12,400 for the same period; Leatherby, L. (2023). Gaza civilians, under israeli barrage, are being killed at historic pace, *New York Times*, 25 November. https://www.nytimes.com/2023/11/25/world/middleeast/israel‐gaza‐death‐toll.html.

42 These data do not list the number of recipients nor distinguish among payments for death, injuries, or property (Government Accounting Office, *Military Operations, The Department of Defense's Use of Solatia and Condolence Payments in Iraq and Afghanistan*, GAO‐07‐699, 2007. https://www.gao.gov/new.items/d07699.pdf.

43 Baker, J. B. (2007). Medical diplomacy in full‐spectrum operations. *Military Review*, *87*, 67–73.

44 CIVIC, op. cit. note 3; Walerstein, op. cit. note 2; CIVIC, op. cit. note 2.

45 The United Nationsl Office for the Coordination of Humaniarian Affairs. (2024). Hostilities in the Gaza Strip and Israel ‐ Flash update #116.

